# Non-Specific Epileptic Activity, EEG, and Brain Imaging in Loss of Function Variants in *SATB1*: A New Case Report and Review of the Literature

**DOI:** 10.3390/genes15050548

**Published:** 2024-04-25

**Authors:** Flavia Privitera, Stefano Pagano, Camilla Meossi, Roberta Battini, Emanuele Bartolini, Domenico Montanaro, Filippo Maria Santorelli

**Affiliations:** 1 Department of Developmental Neuroscience, IRCCS Stella Maris Foundation, Via dei Giacinti 2, 56128 Pisa, Italy; flavia.privitera@fsm.unipi.it (F.P.); stefano.pagano@fsm.unipi.it (S.P.); camilla.meossi@fsm.unipi.it (C.M.); roberta.battini@fsm.unipi.it (R.B.); emanuele.bartolini@fsm.unipi.it (E.B.); 2 Molecular Medicine, IRCCS Fondazione Stella Maris, Via dei Giacinti 2, 56128 Pisa, Italy; 3 Medical Genetics, Residency Program, Federico II University, Via S. Pansini 5, 80131 Naples, Italy; 4 Department of Clinical and Experimental Medicine, University of Pisa, 56126 Pisa, Italy; 5 Tuscany PhD Program in Neurosciences, 50139 Florence, Italy; 6 U.O.S. Dipartimentale e Servizio Autonomo di Risonanza Magnetica, IRCCS Stella Maris Foundation, 56128 Pisa, Italy; domenico.montanaro@fsm.unipi.it

**Keywords:** *SATB1*, WES, phenotype–genotype association, missense variants, premature termination variants

## Abstract

*SATB1* (MIM #602075) is a relatively new gene reported only in recent years in association with neurodevelopmental disorders characterized by variable facial dysmorphisms, global developmental delay, poor or absent speech, altered electroencephalogram (EEG), and brain abnormalities on imaging. To date about thirty variants in forty-four patients/children have been described, with a heterogeneous spectrum of clinical manifestations. In the present study, we describe a new patient affected by mild intellectual disability, speech disorder, and non-specific abnormalities on EEG and neuroimaging. Family studies identified a new de novo frameshift variant c.1818delG (p.(Gln606Hisfs*101)) in *SATB1*. To better define genotype–phenotype associations in the different types of reported *SATB1* variants, we reviewed clinical data from our patient and from the literature and compared manifestations (epileptic activity, EEG abnormalities and abnormal brain imaging) due to missense variants versus those attributable to loss-of-function/premature termination variants. Our analyses showed that the latter variants are associated with less severe, non-specific clinical features when compared with the more severe phenotypes due to missense variants. These findings provide new insights into *SATB1*-related disorders.

## 1. Introduction

The special AT-rich sequence-binding protein 1 gene (*SATB1*, chromosome 3, MIM#602075) encodes a T cell-enriched transcription factor and chromatin organizer essential for controlling genes involved in T-cell development and activation [1]. Although several studies identify *SATB1* as a promoter of tumor cell growth and an inhibitor of apoptosis [2], large exome sequencing studies, integrated with statistical analyses, showed the gene to be potentially involved in brain development; the identification of de novo variants in large cohorts of subjects with neurodevelopmental disorders confirmed its contribution to the pathogenesis of these conditions [3,4,5]. *SATB1* is currently associated with developmental delay with dysmorphic facies and dental anomalies (DEFDA, MIM#619228) and with the Den Hoed-de Boer-Voisin syndrome (MIM#619229), two autosomal dominant disorders characterized by variable facial dysmorphisms, global developmental delay, poor or absent speech, altered electroencephalogram (EEG), and brain abnormalities on imaging. Patients harboring missense variants, usually affecting the two functional domains Crystal Structure of the Ubiquitin-like Domain, (namely, CUT1 and CUT2), seem to show more severe phenotypes characterized by intellectual disability (ID), speech disorder, recurrent pharmacoresistant epileptic encephalopathy, hypsarrhythmia, ventricular enlargement with cortical atrophy, and incomplete myelination [4]. Conversely, Loss-of-Function (LoF)/Premature Termination Variants (PTVs) that escape nonsense-mediated mRNA decay and are mainly located in the C-terminus homeobox domain are associated with milder phenotypes often consisting of mild/severe ID, speech disorders, no epileptic activity or seizure-like activity, and normal brain imaging [4]. In vitro functional studies have demonstrated that missense variants can cause stronger chromatin binding and increase transcriptional repression, while LoF/PTVs are mislocalized in the cell nucleus and may have variable effects on *SATB1* transcriptional activity [4]. 

Because of the relatively limited available information on patient follow up and treatment, we aimed to better characterize *SATB1*-related conditions. In the present study, we report a new patient affected by mild ID, speech disorder, EEG abnormalities, and cortical malformation in whom we identified a new de novo frameshift variant in *SATB1*, never previously described in the literature. We set out to compare this case with published patients harboring LoF/PTVs in the homeobox domain or surrounding area, and also with patients harboring missense variants in the CUT1/CUT2 domains, in order to better define the genotype–phenotype associations and show how clinical features can be topologically related to protein domains.

## 2. Materials and Methods

### 2.1. The Patient

The patient’s blood and signed informed consent document were sent to the clinical laboratory of Molecular Medicine, IRCCS Fondazione Stella Maris (Pisa, Italy). Genetic counseling was performed, and data on family history and clinical and dysmorphic features were collected. All the members of the family trio gave their written informed consent to the study, in accordance with the Declaration of Helsinki. Genomic DNA was isolated and extracted from EDTA peripheral blood samples using a MagCore HF16 extractor (Diatech Lab Line, Jesi, Italy), in accordance with the manufacturer’s instructions. DNA quantity was estimated using an Implen NanoPhotometer P360 v.2.0.0. (Implen, Munich, Germany).

### 2.2. Genetic Testing

Extracted DNA was studied with a 150 bp paired-end protocol on a NovaSeq6000 sequencer (Illumina Inc., San Diego, CA, USA). Whole-exome sequencing (WES) was performed, and DNA libraries were prepared for each DNA sample using the SureSelect Human All Exon V7 system (Agilent Technologies, Santa Clara, CA, USA) in accordance with the manufacturer’s instructions. About 98% coverage of the mappable target >20× was taken as the quality criterion. Reads were mapped against the hg19 reference genome with the Burrows-Wheeler aligner [6], and variants were called with HaplotypeCaller from the GATK suite v.4.0 (Broad Institute, Cambridge, MA, USA). Exome data were analyzed using the enGenome-eVai (v.3.1) and Integrative Genomic Viewer (http://igv.org/, accessed on 10 February 2024) tools. 

Variants were prioritized according to the following criteria: rare variants (minor allele frequency < 0.01), number of homozygotes = zero according to the gnomAD database (https://gnomad.broadinstitute.or/, accessed on 10 February 2024), and clinical data received after genetic counseling. To prioritize variants, we selected the following curated virtual panels of phenotype-related genes from the Genomics England PanelApp (https://panelapp.genomicsengland.co.uk/): ID (v.5.0, 22 March 2023, 2679 genes); early onset or syndromic epilepsy (v.4.0, 22 March 2023, 844 genes); cerebral malformation (v.10.1, 22 March 2023, 158 genes). Only variants occurring in “green” (diagnostic evidence level) genes were considered [7], while synonymous and deep intronic variants were excluded. The following databases were also consulted, all on 15 February 2024 for classification and interpretation of variants: the American College of Medical Genetics guidelines [8], the NCBI dbSNP (https://www.ncbi.nlm.nih.gov/snp/), ClinVar (https://www.ncbi.nlm.nih.gov/clinvar/), LOVD v.3.0 (Leiden Open Variant Database, https://www.lovd.nl/), Franklin by Genoox (https://franklin.genoox.com/clinical-db/home), Mobi Details (https://mobidetails.iurc.montp.inserm.fr/MD/), VarSome (https://varsome.com/), Mastermind (https://mastermind.genomenon.com/), and SFARI gene (Simon’s Foundation Autism Research Initiative, https://gene.sfari.org).

## 3. Results

### 3.1. Clinical Phenotype

**Figure 1:**
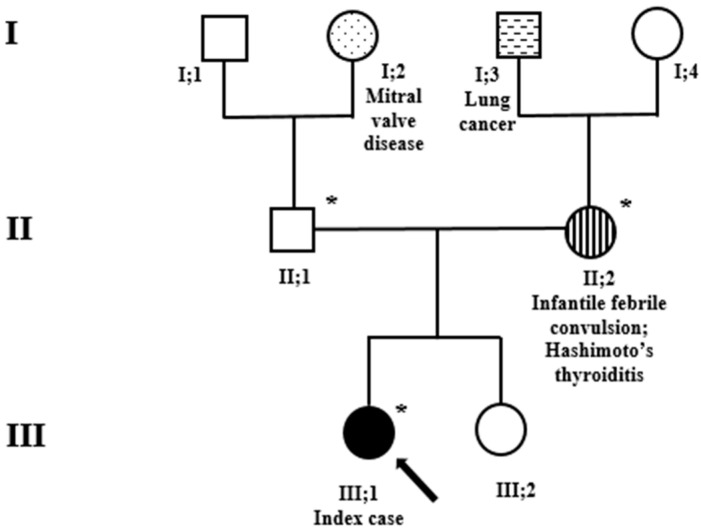
Family pedigree. Exome sequencing analysis was performed in the index case (black arrow) and her parents. *, sampled individuals. Circles = females; squares = males; black = family members affected by variants in the *SATB1* gene. Stripes, hatches and dots: other clinical and relevant manifestations.

The index case was identified in a large study (ongoing) addressing the diagnostic yield of the exome in the routine clinical setting of children with neurodevelopmental disorders. We first saw the patient at the age of 8 years when she was referred with possible language delay. She was the firstborn child of unrelated parents; the family history was remarkable for lung cancer in the maternal grandfather, and mitral valve disease in the paternal grandmother (Figure 1). Whilst the 43-year-old father and the younger (6-year-old) sister are healthy, the 44-year-old mother reported that she had experienced a febrile convulsion at the age 2 of years and been diagnosed with Hashimoto’s thyroiditis at the age of 14 years. She also reported a miscarriage during the second trimester of a previous pregnancy. The patient was born at term by C-section performed for suspected macrocrania at 38 weeks of gestation. The prenatal and perinatal periods were unremarkable. The growth parameters at birth were the following: weight 2.96 kg, length 51 cm, and occipital frontal circumference (OFC) 34 cm. A brain ultrasound performed due to suspected delayed closure of the anterior fontanel was normal. 

The patient reached the developmental milestones on time, achieving independent walking at 13 months without having crawled. Babbling and first words occurred before the age of 12 months. A speech arrest at around age 18 months was reported. 

The patient attended nursery school, where she had socialization difficulties; she was irritable and had disturbed nocturnal sleep, although this resolved itself after a few months. A first examination at the age of 2 years with Griffiths Mental Development Scales, audiometric test and EEG suggested she should start speech therapy. A polysomnography for suspected sleep apnea reported “short hypopnea sequences with low desaturation”. 

**Figure 2:**
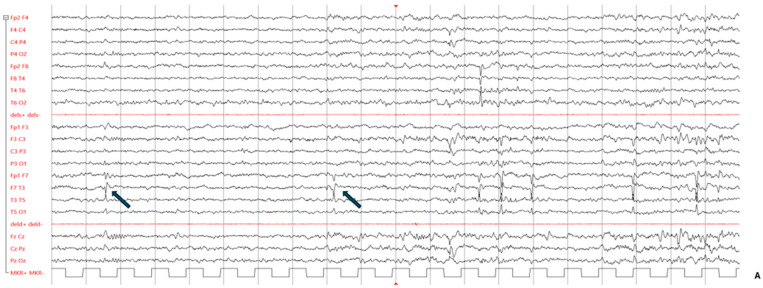

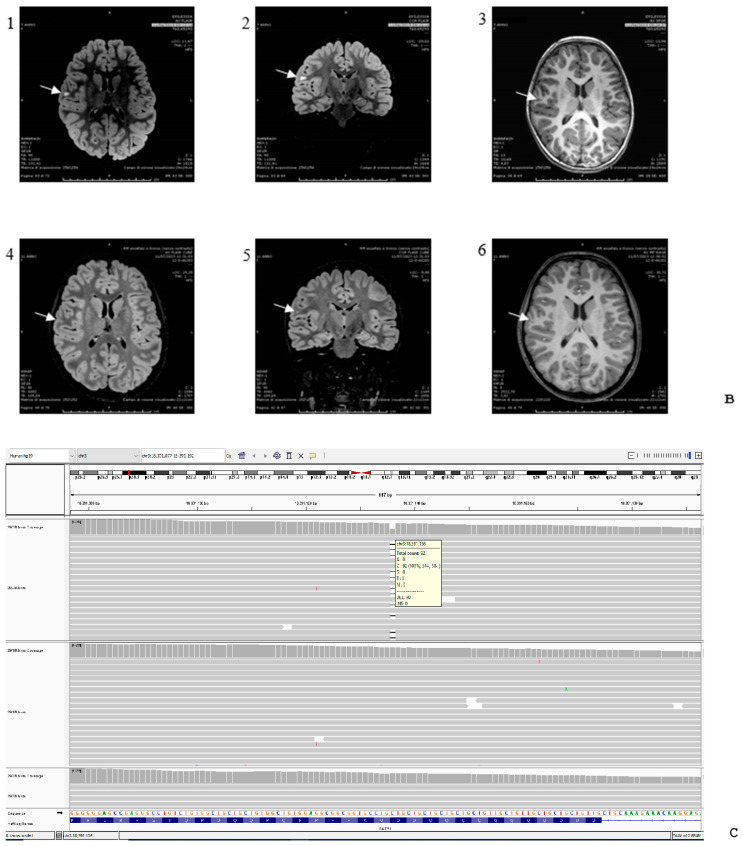

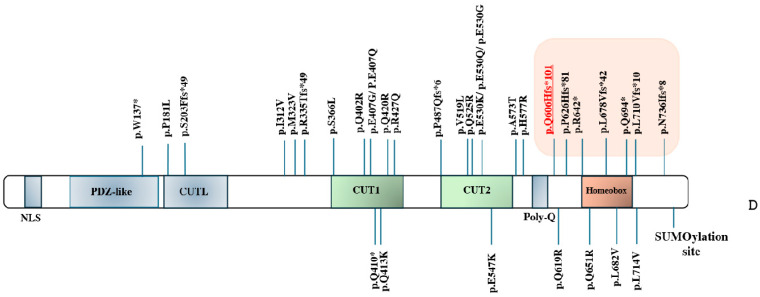
(**A**) Patient’s abnormal EEG. Blue arrows indicate bilateral sharp wave as well as spike-and-wave discharges in the temporal regions (right > left), enhanced in sleep. (**B**) MRI examinations were performed at age 7 (panels 1, 2, 3) and 11 years (panels 4, 5, 6). T2-FLAIR images are shown on axial (panels 1, 4) and coronal plane (panels 2, 5), and T1-weighted images are shown on axial plane (panels 3, 6). Focal hyperintensity was seen on T2-FLAIR images (panel 1, 2) and as mild hypointensity on T1w (panel 3) in the right subcortical frontal white matter (arrows), but drastically reduced at follow-up (panels 4–6). (**C**) Genomic display on IGV of the *SATB1* variant (chromosome 3, cytoband p24.3) in the family trio [coverage (alternate/reference): 142 (70/72)]. From the top to the bottom: case index, father, mother. The variant refers to human genome assembly GRCh37/hg19. (**D**) Schematic representation of the *SATB1* gene (MIM#602075, chromosome 3, NM_002971.4/NM_001131010.4) with the CUT1/CUT2 domains in green and the Homeobox domain in orange. The variants covered by this study are in orange highlighted in the box on the right; the new de novo frameshift variant described in our child is in red underlined. All the other variants have been previously described [4,9,10].

At age 3 years, the child underwent a first EEG which showed epileptiform discharges in the central and right temporal areas. A follow-up examination one year later confirmed the EEG abnormalities, with no focal seizures; for this reason, no antiepileptic treatment was suggested. A new EEG performed at the age of 6 years showed continuous spike-and-wave discharges during sleep in the bilateral temporal regions. On this basis, a short course of levetiracetam was prescribed. Further EEG examinations performed to date have confirmed the presence of bilateral sharp waves as well as spike-and-wave discharges in the temporal regions (right > left), enhanced during sleep (Figure 2A).

A first magnetic resonance imaging (MRI) examination at the age of 5 years (Figure 2B) showed a focal hyperintense signal in the T2 and T2-FLAIR sequences, hypointense on T1-weighted images in the right frontal supra-opercular subcortical region. The size of this signal alteration was clearly reduced on a 3T brain MRI performed six years later, leading to the hypothesis of hamartoma (Figure 2B). 

At the age of 9 years, the patient attended school with support, produced simple sentences and had significant limitations in basic activities of daily living. She presented the following minor dysmorphic features: mild macrocephaly, arched upper lip, widely spaced teeth, and a hyperchromic spot in the glabellar region. 

At the age of 13 years of age, her weight was 48 kg, her height was 157 cm, and her OFC was 56.5 cm. No eating or sleeping difficulties were reported, nor any difficulties at school. Scoliosis and the use of orthoses were reported.

### 3.2. Genetic Analyses

Karyotype, array Comparative Genomic Hybridization (aCGH), and methylation test of the 15q11 region were all normal. Targeted sequencing of multigene panels in next-generation sequencing for genes associated with cortical dysplasia highlighted the presence of the c.151A>G(p.(Met51Val)) variant in the *DCX* gene (MIM*300121) and c.1186G>A(p.(Val396Ile)) in the *PAFAH1B1* gene (MIM*601545), both paternally inherited.

Whole-exome sequencing performed on the family trio showed the variants listed in Supplementary Table S1. Among them, only the heterozygous variant, c.1818delG (p.(Gln606Hisfs*101)) in the *SATB1* gene, emerged as the likely candidate associated with the patient’s phenotype. Trio analysis showed that the variant arose de novo in the family (Figure 2C).

## 4. Discussion

Patients harboring *SATB1* variants are rare, and precise genotype–phenotype correlations are yet to be defined. The most detailed associations have been provided by Den Hoed et al. [4], who collected clinical findings from a group of 42 patients harboring rare variants in *SATB1* and performed functional analyses, assessing the consequences in terms of cellular localization, transcriptional activity, overall chromatin binding, and dimerization capacity. This allowed them to classify pathogenic missense variants located in the CUT1 and CUT2 domains as generally associated with more severe phenotypes, and pathogenic LoF/PVTs located in the homeobox domain, and its surrounding area as associated with less severe manifestations [4]. 

In the present study, we described a new patient affected by ID, speech disorder, and EEG/MRI abnormalities, who harbors a new de novo PVT in *SATB1* near to the Homeobox domain. The presence of this variant, not previously described in the literature, suggested a possible new diagnosis of Den Hoed–de Boer–Voisin syndrome (MIM#619229). We then compared the clinical manifestations in patients carrying missense variants and patients with LoF/PVTs ones, to better delineate the phenotypes most frequently associated with these different mutations.

To date, few LoF/PVTs have been described in *SATB1*; from a literature review [4,5,9,10], with the addition of the new c.1818delG variant described in the present study, only seven variants in eight patients have been described (Figure 2D). Table 1 recapitulates all the clinical features of all seven patients. Five of them (including our case) had a variant arising de novo; transmission from an affected father occurred in patient 4, and from an unaffected mother in patient 7; uncertain inheritance was reported for patient 5. Family history showed seizures on the maternal side of the family in patient 2; juvenile epilepsy and neurodevelopmental delay were reported in the family history of patient 4 and tremors in that of patient 7, in both cases on the paternal side (Table 1).

As regards the clinical features, ID, epilepsy, abnormal EEG and altered MRI were reported in 4/8 patients (50%); developmental and motor delay in 8/8 (100%); speech delay in 7/8 (88%); dystonia and sleep disturbances in 1/8 (13%); hypotonia in 3/8 (38%); ataxia and behavioral disturbances in 2/8 (16%); facial dysmorphisms in 6/8 (75%); and dental abnormalities in 5/8 (63%). 

In the present study, we compared the frequency of clinical features reported, respectively, in patients with missense variants, in patients with LoF/PVTs, and in our new case. In most of the cases with LoF/PVTs, we noted absent or mild epileptic activity, non-specific EEG and brain imaging, and no association with epilepsy [4,9] or seizure-like activity [4]. In a single infant there was occurrence of major seizures [10]. However, patients showed abnormal EEGs with bifrontal spikes; sharp, sharp slow, spinous slow waves in the bilateral occipital areas [10]; epileptiform discharges in the centro-temporal regions, enhanced in sleep; and occasional theta slowing in the left and right temporal and posterior quadrants, at times with occasional diffuse theta slowing with occipital predominance, features in part also seen in our patient (Table 2). Like our patient, the LoF/PVT cases usually showed an abnormal subcortical white matter hyperintense signal on T2-FLAIR sequences, few punctiform frontal white matter changes, mild bifrontal white matter volume loss, mild prominence of the frontal horns and bodies, mild symmetric prominence of the subarachnoid fluid, and normal MRI (Table 2). These observations reinforce the suggestion that PTVs in *SATB1* are rare and that patients can be missed because of the relatively nonspecific features found in children with neurodevelopmental disorders. This is a further element in favor of the use of trio WES analysis as a first-tier diagnostic test in patients with non-syndromic neurodevelopmental disorders.

WES analysis on the present study also detected other missense heterozygous variants in genes potentially associated with neurodevelopmental disorders (Table S1), including *SRCAP* (MIM*611421), associated with Developmental delay, hypotonia, musculoskeletal defect and behavioral abnormalities (MIM#619595) and Floating-Harbor syndrome (MIM#136140), and *CACNA1A* (MIM*601011), associated with autosomal dominant conditions such as Developmental and epileptic encephalopathies (MIM#617106), episodic ataxia (MIM#108500) and Spinocerebellar ataxia type 6 (MIM#183086). WES studies also confirmed the two variants previously highlighted by NGS targeted gene panel in *PAFAH1B1* (MIM*601545) and *DCX* (MIM*300121), both paternally inherited. Both these genes are associated with Lissencephaly 1 and Subcortical Laminar Heterotopia, with autosomal dominant inheritance for *PAFAH1B1* (OMIM#607432), and with X-Linked inheritance for *DCX* (MIM#300067). Truncating variants in the *SRCAP* gene have been indeed identified in distinct neurodevelopmental disorder characterized by developmental delay with or without ID, behavioral and psychiatric abnormalities, non-specific facial features, musculoskeletal abnormalities, and hypotonia [11]; LoF and missense variants were also identified in patients with autism [12,13,14,15]. Variants in the *CACNA1A* gene were identified in affected individuals from four unrelated families presenting with a spectrum of cognitive impairment including intellectual disability, executive dysfunction, ADHD and/or autism, as well as childhood-onset epileptic encephalopathy with refractory absence epilepsy, febrile seizures, downbeat nystagmus and episodic ataxia, and damaging missense and likely loss-of-functions in *CACNA1A*, many of which were de novo in origin, have subsequently been identified in individuals presenting with similar phenotypes [16,17,18,19,20].

Although we cannot completely exclude that the aforementioned variants contribute to the final phenotype, we observed that they were VUS inherited from a healthy parent with a normal neurological development. Thus, their overall contribution remains speculative at this stage. 

Regarding the new c.1818delG(p.(Gln606Hisfs*101)) in the current study, it is unlikely it leads to mRNA degradation by Nonsense Mediated Decay (NMD) applying the bioinformatics tolls used by others [4,21], though we did not test this functionally. In silico we predicted a shorter protein than the wild type, with modification at the C-terminus end. The presence in literature of additional variants localized downstream in patients with mild epileptic activity and non- specific brain imaging also suggests correlation of the new variant with the patient’s phenotype.

## 5. Conclusions

In conclusion, our study provides new clinical and molecular information useful for the management of patients with *SATB1* variants. Patients with LoF/PVTs are still few in number, and further investigations are needed to better define main clinical features and their progression, considering also antiepileptic drugs as possible therapies [10]. This report adds a new gene variant to those already known to be associated with *SATB1*-related disorders. Future reports of similar cases might help to drive more precise monitoring of disease progression in this syndrome.

## Figures and Tables

**Table 1:** Phenotypes described in the literature and in the present study, associated with LoF/PVTs variants in the Homeobox domain and its surrounding area of the protein encoded by the SATB1 gene. n.a.: not available. +, present; -, absent. y.o.: years old; CTG: cardiotocography. OFC: Occipital Frontal Circumference. ID: Intellectual Disability. EEG: electroencephalogram. *: Patients 4 and 5 are father and daughter.

	Genotype of *SATB1*	Patient 1 Present Study: c.1818delG (p.(Q606Hfs*101))	Patient 2 c.1877delC (p.(P626Hfs*81)) [4]	Patient 3 c.1924C>T (p.(R642*)) [9]	Patient 4* (Father) c.2032_2033delCT (p.(L678Vfs*42)) [4]	Patient 5* (Daughter) c.2032_2033delCT (p.(L678Vfs*42)) [4]	Patient 6 c.2080C>T (p.(Q694*)) [4]	Patient 7 c.2207delA (p.(N736Ifs*8)) [4]	Patient 8 c.2128_2129del (p.L710Vfs*10)) [10]
Clinical features									
Family history		Negative	Familiarity for seizure on the maternal side (grandfather and cousin)	Negative	Negative	Father with juvenile epilepsy and neurodevelopmental delay; mother with obesity	Negative	Tremors on the paternal side; mother in good health	Negative
Inheritance of the variant		de novo	de novo	de novo	Unknown	Paternally inherited	de novo	Maternally inherited	de novo
Other genetic findings		See Supplementary Table S1	Negative	Karyotype 47, XXX	Negative	Negative	Heterozygous, de novo variant in *NAGA* (NM_000262.2) c.791_792del; (p.(E264Afs*72)), OMIM# 609241; negative CGH-array.	Heterozygous, de novo variant in *ARHGAP35* (NM_004491.4); c.493 G>T (p.(D165Y)))	Negative
Abnormalities during pregnancy		-	-	-	-	-	+, placenta previa and bleeding	-	-
Abnormalities during delivery		+, cesarean section due to macrocephaly at 38 weeks of gestation	+, apneic episode at 12 h of life, requiring intubation and phenobarbital	-	-	+, cesarean section due to pathological CTG	+, ruptured placenta at 27 weeks leading to emergency cesarean section delivery	+, perinatal distress with low Apgar scores	-
Birth weight		2.96 kg	3.09 kg	n.a.	n.a.	3.64 kg	1.25 kg	3.2 kg	n.a.
OFC at birth		34 cm	n.a.	n.a.	n.a.	33 cm	n.a.	34 cm	n.a.
ID		+	+, mild	+	-	n.a.	n.a.	n.a.	+
Developmental delay		+	+, mild	+, mild, special education	+	+, severe combined disorder	+	+	+
Motor delay		+	+, toe walking in early childhood, physiotherapy and occupational therapy	+	+	+	+	+	+
Speech delay		+	+, speech therapy	+	-	+	+	+	+
Behavioral disturbances		-	-	+	-	-	-	-	+
Sleep disturbances		-	n.a.	-	-	-	-	-	+
Epilepsy		+	+, seizure-like activity at 7, 9 and 11 y.o.; resolved in adulthood	-	+, at 3 y.o., treated with anticonvulsant until 6 y.o. 2 further episodes of seizures at 10 and 15 y.o., due to photosensitivity while playing video games	-	-	-	+, major seizure lasted five minutes at 2 months
EEG abnormalities		+, epileptiform discharges in the centro-temporal regions, enhanced in sleep	+, abnormal EEGs with bifrontal spikes	-	-	-	+, occasional theta slowing in L and R temporal, and posterior quadrants, at times with occasional diffuse theta slowing with occipital predominance suggesting the presence of diffuse and focal cortical dysfunction with encephalopathy. No apparent interictal discharges. Events of concern were captured and found to be nonepileptic in nature.	-	+, sharp, sharp slow, spinous slow waves in the bilateral occipital areas, more prominent on the right side
Brain imaging		+, abnormal subcortical white matter hyperintense signal on T2-FLAIR sequences	+, normal at 7 y.o.; prominent cisterna magna at 9 y.o.	+, few punctiform frontal white matter changes	Normal	n.a.	+, mild bifrontal white matter volume loss with mild prominence of frontal horns and bodies of both lateral ventricles, mild symmetric prominence of subarachnoid fluid over frontal convexities and along anterior interhemispheric fissure	Normal	Negative
Hypotonia		-	-	-	-	+, truncal	+	-	+
Dystonia		-	n.a.	+	n.a.	n.a.	n.a.	n.a.	n.a.
Ataxia		-	-	+	-	-	-	+	-
Facial dysmorphisms		+, minor: mild macrocephaly, arched upper lip, hyperchromic spot in the glabellar region	+	+	-	-	+, tall prominent forehead, medial eyebrow flare, bulbous nasal tip, deep, short philtrum, prominent chin	+, subtle	+, subtle
Dental abnormalities		+, widely spaced teeth	+, missing molars	-	-	-	+, small widely spaced teeth	+, enamel dysplasia	+, widely spaced teeth

**Table 2:** Comparison of epileptic activity, EEG and MRI abnormalities caused by variants in the CUT1-CUT2 domains versus those ones localized in the Homeobox domain of the SATB1 gene: present study and review of the literature. (PS): present study.

Clinical Findings	CUT1-CUT2 Domains (Missense Variants) [4]	Homeobox Domain (LoF/PVTs) ([4,9,10], [PS])
Epileptic manifestations	Recurrent pharmacoresistant epileptic encephalopathy, diagnosed in infancy (<1 year old) [4];	No epilepsy [3,9] or seizure-like activity [4];
	multiple absences [4];	A single infantile major seizure [10].
	uprolling of eyeballs with loss of consciousness [4];	
	tonic/clonic movement of limbs [4];	
	tonic/myoclonic seizures [4];	
	status epilepticus [4].	
EEG abnormalities	Hypsarrhythmia [4];	Abnormal EEGs with bifrontal spikes [4];
	Lateral multifocal discharges [4];	Epileptiform discharges in the centro-temporal regions, enhanced in sleep (PS);
	Focal epileptiform activity over right occipital region with asymmetric photic driving response [4];	Occasional theta slowing in left and right temporal and posterior quadrants, at times with occasional diffuse theta slowing with an occipital predominance suggesting presence of diffuse and focal cortical dysfunction with encephalopathy [4];
	Multifocal mainly bi-frontocentral epileptiform activity, more frequently occurring during sleep than during wakefulness [4];	Sharp; sharp slow, spinous slow waves in the bilateral occipital areas, more prominent on the right side [10].
	Poor organization, slow background consistent with at least a moderate encephalopathy in addition to polyspike and slow wave discharge. During sedation: Poorly organized posterior basic rhythm, poorly developed theta with very prominent beta activity superimposed. During sleep: No well-formed sleep architecture. Intermittent periods of rhythmic bifrontal alpha waves. Bursts of high amplitude polyspike and wave discharges, occurring as often as every 3–4 s. Electrographic onset, characterized by bifrontal alpha rhythmic activity, precedes the myoclonic jerks by several seconds [4];	
	Focal epileptiform activity [4];	
	Tonic seizure activity [4].	
MRI findings	Global supra- and subtentorial brain atrophy [4];	Abnormal subcortical white matter hyperintense signal on T2-FLAIR sequences (PS);
	White matter hyperintensities [4];	Few punctiform frontal white matter changes [9];
	Ventricular enlargement with cortical atrophy [4];	Mild bifrontal white matter volume loss [4];
	Incomplete myelination [4];	Mild prominence of frontal horns and bodies [4];
	Subarachnoid space enlargement [4];	Mild symmetric prominence of subarachnoid fluid [4];
	Cerebellar atrophy [4].	Normal MRI [4,10].

## Data Availability

The original contributions presented in the study are included in the article/Supplementary Material, further inquiries can be directed to the corresponding author.

## Supplementary Materials

The following supporting information can be downloaded at: https://www.mdpi.com/article/10.3390/genes15050548/s1, Table S1: WES results in the family trio.
