# Supplementary material for: Non-Specific Epileptic Activity, EEG, and Brain Imaging in Loss of Function Variants in SATB1: A New Case Report and Review of the Literature
Source: Genes (Basel). 2024 Apr 25;15(5):548. doi: 10.3390/genes15050548 (PMC11120869; doi:10.3390/genes15050548)
Supplement: Supplementary file 1 [file genes-15-00548-s001.zip › genes-2976310-supplementary.pdf]

Supplementary, Table S1. WES results emerged from the family trio. Variants all refer to the Human Genome Assembly GRCh37/hg19, and they are all described in a heterozygous status.

Gene	Transcript	HGVS_coding (protein)	Inheritance	Allele frequency	ACMG Classification[7]
SATB1	NM_002971.6 ENST00000417717	c.1818delG(p.(Gln606fs*101))	*de novo*	0%	Likely Pathogenic
SRCAP	NM_006662.3 ENST00000262518	c.3640A>T(p.(Asn1214Tyr))	paternally	0.000795%	VUS
CACNA1A	NM_001127222.2 ENST00000360228	c.6125C>T(p.(Thr2042Met))	maternally	0.003678%	VUS
PAFAH1B1	NM_000430.4 ENST00000397195	c.1186G>A(p.(Val396Ile))	paternally	0.001415%	VUS
DCX	NM_000555.3 ENST00000338081	c.151A>G(p.(Met51Val))	paternally	0.024542%	Benign

VUS: Variants of Uncertain Significance. Metrics such as Combining Annotation-Dependent Deletion (CADD-Phred) for scoring the deleteriousness of variants (>20), Rare Exome Variant Ensemble Learner (REVEL) to distinguish pathogenic from rare neutral variants with allele frequencies <0.5% (<0.2: benign, >0.5: damaging), and MoBiDiC Prioritization Algorithm (MPA: raw score 1-10; 10: high impact) were considered to choose variants of clinical interest.
